# Effect of surgical timing in 23-g pars plana vitrectomy for primary repair of macula-off rhegmatogenous retinal detachment, a retrospective study

**DOI:** 10.1186/s12886-022-02364-4

**Published:** 2022-03-25

**Authors:** Omar Elghawy, Ryan Duong, Amen Nigussie, Joseph D. Bogaard, James Patrie, Yevgeniy Shildkrot

**Affiliations:** 1 University of Virginia School of Medicine, Charlottesville, VA USA; 2 Department of Ophthalmology, University of Virginia, Charlottesville, VA USA; 3 Department of Public Health Sciences, UVA, Charlottesville, VA USA

**Keywords:** Macula-off, Pars plana vitrectomy, Rhegmatogenous retinal detachment, Duration, Visual outcomes, Complications

## Abstract

**Background:**

Rhegmatogenous retinal detachment (RRD) is a common, potentially blinding ocular pathology that is considered a surgical emergency. Macular involvement has been identified as a major negative prognostic indicator for visual recovery after RRD correction. It is not currently clear whether early intervention improves visual outcomes, and in practice, there are potential disadvantages to performing early surgery for fovea-involving RRD. Such disadvantages include inadequate assessment of coexisting comorbidities, increased rate of complications related to poorly trained staff or tired surgeons, and anesthetic risk.

**Methods:**

A single-center, retrospective, cohort study of patients who underwent repair of macula-involving rhegmatogenous retinal detachment at the University of Virginia was performed. Variables collected included patient demographics, ocular history, clinical characteristics, and post-operative complications. Patients were excluded if they had a history of congenital or acquired pathology with an effect on visual function, bilateral or repeat rhegmatogenous detachment, age less than 18 years, follow up duration less than 6 months, or if they were repaired using scleral buckle, pneumatic retinopexy, 25- or 27-gauge pars plana vitrectomy, or any combination of these techniques. A multivariate regression model was used to compare overall outcomes such as post-operative visual acuity, intra-ocular pressure, retina attachment status, and complications among patients of differing timing of surgical repair. These analyses were adjusted for clinical factors known or considered to be associated with worse prognosis in rhegmatogenous retinal detachment.

**Results:**

A total of 104 patients undergoing 23-gauge vitrectomy for repair of macula involving rhegmatogenous retinal detachments were included in this study with mean follow up period 17.9 ± 14.1 months. Early surgical repair (< 48 h) was pursued in 26 patients, moderately delayed surgical repair (3–7 days), was performed in 29 patients and late surgical repair (> 7 days) in 49 patients. Our analysis showed no difference in post-operative visual acuity between patients with detachments undergoing early versus moderately delayed repair of RRD. However, mean visual acuity differed between patients undergoing early versus late repair at 3, 6, and 12 months. No significant difference was observed in post-operative complications between the three surgical timepoints including cataract formation, development of glaucoma and re-detachment rate. Use of 360 laser was found to be protective against re-detachment overall (OR 6.70 95% CI 1.93–23.2).

**Conclusions:**

These findings indicate that a moderate delay of 3–7 days from symptom onset for repair of macula-involving retinal detachment may be a safe approach as there are no differences in terms of visual acuity or post-operative complications compared to early repair within 48 h. Delaying surgery for > 7 days however is not recommended due to the loss of recovery of visual acuity observed in this study. Use of 360 laser may prevent risk of re-detachment after primary repair.

**Supplementary Information:**

The online version contains supplementary material available at 10.1186/s12886-022-02364-4.

## Background

Rhegmatogenous retinal detachment (RRD) is a potentially blinding ocular pathology that occurs when the neurosensory retina is separated from the underlying retinal pigmented epithelium [1]. The condition is relatively common, with an incidence as high as 17.9 per 100,000 people per year in the United States [2]. Current treatment centers around reattachment of the retina via pneumatic retinopexy, scleral buckling, or vitrectomy which have varying levels of success [3]. Pars plana vitrectomy (PPV) has been growing as the first-choice treatment method for RRD in recent years [4]. Vitrectomy has some of the highest anatomical success rates among all procedures with an overall mean postoperative reattachment rates as high as 93.3%, according to a study evaluating all forms and severities of primary rhegmatogenous retinal detachment [5]. Despite these successful anatomical outcomes, functional outcomes are largely unpredictable and highly individually variable [3–6].

Several variables including extent of retinal detachment, previous intraocular surgery, high myopia, and preoperative best-corrected visual acuity have been associated with worse functional outcomes after surgical correction of RRD [7, 8]. Macular detachment has also been identified as a major negative prognostic indicator for visual recovery after RRD correction, and standard practice utilizes macular inclusion as a measurement for determining surgical urgency [6].

Time until surgery is one of the most important and modifiable prognostic factors for fovea-involving RRD [9, 10]. However, the precise amount of tolerable delay in fovea-involving RRD remains unclear and controversial. While previous studies have concluded functional damage following a macular detachment event is permanent, others have shown that poorer visual outcomes are associated with longer durations of preoperative macular detachment [8–10]. Mechanistically, studies in animal and cell models have demonstrated that photoreceptor cell death is immediately induced, as early as 12 h after the RD event, and peaks after 2 or 3 days, however, the photoreceptor degeneration processes are complex and are currently not fully understood [11–14]. This may suggest that fovea-involving RRD outcomes would be best if treated as soon as possible, but this has only been demonstrated in a small minority of clinical studies [15].

In practice, there are potential disadvantages to performing early surgery for fovea-involving RRD, such as inadequate assessment of comorbidities or increased complications related to poorly trained staff or tired surgeons. Anesthetic risk that may limit outcomes with early surgery explain this discrepancy. In addition, increased cost and utilization of resources should be considered when deciding to operate emergently for fovea involving RRD. These disadvantages must be weighed against the risks of delaying surgery such as progression of the detached retina and development of proliferative vitreoretinopathy. Thus, the recommended maximum lag time between fovea detachment and repair remains poorly defined and variably reported in the literature, between 1 and 10 days [9, 10, 15, 16].

Furthermore, the ability to reliably and consistently date the time of fovea detachment remains a limitation given the relatively the small number of prior studies evaluating delay in repair for fovea-involving RRD. As these studies have defined duration of detachment based on reported symptoms such as loss of central vision, a lack of objective methodology for dating foveal detachment limits the ability to compare these studies and draw general conclusions.

In the absence of objective methodology to date the time of foveal detachment clinically, increased outcomes analysis is needed to answer the important clinical question—how urgently should a fovea-involving RRD be treated? This study aims to add to the limited literature and provide guidance regarding the optimal timing for operating on fovea-involving RRD by analyzing foveal-involving rhegmatogenous retinal detachments repaired via 23-gauge (23-g) pars plana vitrectomy. We seek to address optimal surgical timing by comparing repair outcomes based on symptomatic duration of macular detachment and defining an amount of tolerable delay in surgery for fovea involving RRD. We hypothesize that earlier repair is associated with better outcomes and fewer complications.

## Methods

A single-center, retrospective, cohort study of 754 consecutive patients who had underwent retinal surgery at the University of Virginia between July 1, 2012, and July 1, 2020, was conducted. Data collection and analysis were performed in concordance with the University of Virginia Institutional Review Board. This research adhered to the tenets of the Declaration of Helsinki and was conducted in accordance with regulations set forth by the Health Insurance Portability and Accountability Act.

### Data collection

Variables collected included patient demographics, ocular history, clinical characteristics, surgical and pharmacological treatments received, and post-operative complications. Patients were excluded if they did not have a fovea-involving rhegmatogenous retinal detachment, had a history of congenital or acquired pathology with an effect on visual function, had a bilateral rhegmatogenous detachment, if their rhegmatogenous retinal detachment was a repeat detachment, if the patient was under 18 years of age, if follow up duration was less than 6 months, or if they were repaired using scleral buckling, 25 or 27 gauge pars plana vitrectomy, pneumatic retinopexy or any combination of these surgical techniques. Consistent with the prior literature, time from reported symptom onset of central or peripheral visual acuity change until operative repair was used to define the duration of detachment. Timing for surgical repair was categorized accordingly as either early repair (symptoms < 48 h), moderately delayed repair (symptoms ≥ 48 h and < 7 days), or late repair (≥ 7 days).

### Statistical analyses

A multivariate regression model was used to compare overall outcomes such as post-operative visual acuity, intra-ocular pressure, retina attachment status, and complications among patients of differing timing of surgical repair. These analyses were adjusted for clinical factors known or considered to be associated with worse prognosis in rhegmatogenous retinal detachment including use of PFO, prior surgery, and increasing number of clock hours detached. Using standardized protocols, the Snellen acuity was obtained and converted to logMAR equivalents. Visual acuity values count fingers (CF), hand motion (HM), light perception (LP), and no light perception (NLP) were converted to 2.0, 2.4, 2.7, and 3.0, respectively [17].

### Data summarization

Empirical categorical scale data was summarized by frequencies and relative frequencies, and empirical continuous scale data was summarized by the mean and standard deviation (SD) of the empirical distribution.

### Pre-operative visual acuity and intraocular pressure (IOP)

Pre-operative visual acuity was analyzed on the logMAR scale. Comparisons of mean pre-operative logMAR between patients with early, moderately delayed, and late surgical repair (symptoms lasting < 48 h, ≥ 48 h and < 7 days, and ≥ 7 days) were conducted via One-way Analyses of Variance (One-way ANOVA). Linear contrasts of the ANOVA least square means were constructed to conduct a global null hypothesis test, where under the null hypothesis it was assumed that all detachment surgical timing groups (symptoms lasting < 48 h, ≥ 48 h and < 7 days, and ≥ 7 days) have the same mean pre-operative logMAR, and to conduct pairwise hypothesis tests, where under the null hypothesis it was assumed that the 2 detachment surgical timing groups (e.g. symptoms lasting < 48 h vs ≥ 48 h and < 7 days) have the same mean pre-operative logMAR. For the global null hypothesis, a *p* ≤ 0.05 decision rule was used as the null hypothesis rejection criterion and for the pairwise null hypothesis tests, a two-sided Bonferroni corrected *p* ≤ 0.05/3 criterion was used as the null hypothesis rejection criterion.

### Post-operative 3-, 6-, and 12-month visual acuity

Post-operative visual acuity was analyzed on the logMAR scale. Comparisons of the 3-, 6-, and 12-month mean logMAR between patients of differing surgical timing groups were conducted via a linear mixed model (LMM). For each assessment time point, a set of linear contrasts of the LMM least square means were constructed to conduct a global hypothesis test, where under the null hypothesis it was assumed that all detachment surgical timing groups have the same mean pre-operative logMAR and to conduct pairwise null hypothesis tests, where under the null hypothesis it was assumed that the detachment surgical timing comparison groups have the same mean logMAR. For the global null hypothesis test, a *p* ≤ 0.05 decision rule was used as the null hypothesis rejection criterion, and for the pairwise null hypothesis tests a two-sided Bonferroni corrected *p* ≤ 0.05/3 decision rule was used as the null hypothesis rejection criterion.

### Final visual acuity

Final visual acuity was analyzed on the logMAR scale. Comparisons of mean pre-operative logMAR between patients of differing surgical timing groups were conducted via analysis of covariance (ANCOVA), where the between group comparisons were adjusted for the length of time until the final visual acuity assessment. Linear contrasts of the ANCOVA least square means were constructed to conduct a global null hypothesis test, where under the null hypothesis it was assumed that all detachment surgical timing groups have the same mean final logMAR, and to conduct pairwise hypothesis tests, where under the null hypothesis it was assumed that the 2 detachment groups (e.g. early repair vs moderately delayed repair) have the same mean final logMAR after adjustment for length of time until the final visual acuity assessment. For the global null hypothesis, a *p* ≤ 0.05 decision rule was used as the null hypothesis rejection criterion and for the pairwise null hypothesis tests, a two-sided Bonferroni corrected *p* ≤ 0.05/3 criterion was used as the null hypothesis rejection criterion.

### IOP analyses

Pre-operative IOP, post-operative 3-, 6-, and 12-month IOP, and final IOP were analyzed using the same analytical methods as were undertaken to analyze pre-operative visual acuity, post-operative 3-, 6-, and 12-month visual acuity, and final visual acuity.

### Complications

Post-operative complications were summarized via frequencies and relative frequencies, and a Pearson exact test was used to compare the complication frequencies between the three different retinal detachment repair groups (symptoms lasting < 48 h, lasting ≥ 48 h and < 7 days, and lasting ≥ 7 days). Logistic regression was used to evaluate the odds for re-detachment as a function of case length and 360 laser usage.

## Results

### Patient characteristics

A total of 104 patients undergoing 23-gauge vitrectomy for repair of rhegmatogenous retinal detachments were included in this study with average case length 109 ± 36.7 min and mean follow up period 17.9 ± 14.1 months. Early surgical repair (retinal detachments symptoms lasting < 48 h) was pursued in 26 patients (25%). Moderately delayed surgical repair (retinal detachment symptoms lasting ≥ 48 h and < 7 days) was performed in 29 patients (28%). Late surgical repair (retinal detachment symptoms lasting ≥ 7 days) was performed in in 49 patients ( mean duration of 32.4 days, median 30 days, (range 10–180 days)). (47%). Tamponade agents for each repair group are detailed in (Supplemental Table 1). Subgroup analysis of patient demographics and medical history identified a higher mean age in patients undergoing early surgical repair (< 48 h) (61.3 ± 15.5) compared undergoing late repair (≥ 7 days) (51.8 ± 18.7) (*p* = 0.02) and more frequently reported prior cataract surgery in patients undergoing early repair (65.4%) compared to moderately delayed (34.5%) and late (26.5%) groups (*p* = 0.032 and *p* = 0.001, respectively). Otherwise, patients of different surgical timing groups did not differ significantly regarding past medical and ocular history (Table 1).

**Table 1:** Characterization of demographics and ocular history among patients with differing surgical repair timing

	Surgical Timing Groups			Overall
	Early Repair (*n* = 26)	Moderately Delayed Repair (*n* = 29)	Late Repair (*n* = 49)	
Demographics				
male	16 (61.5)	15 (51.7)	37 (75.5)	68 (65.4)
female	10 (38.5)	14 (48.3)	12	36 (34.6)
mean age	61.3 ± 15.1*	57.1 ± 12.9	51.8 ± 18.7*	55.6 ± 16.8
Prior surgeries				
vitrectomy	3 (11.5)	1 (3.4)	4 (8.2)	8 (7.7)
glaucoma surgery	2 (100)	0 (0)	3 (50)	5 (41.7)
corneal surgery	3 (11.5)	5 (17.2)	8 (16.3)	16 (15.4)
cataract surgery	17 (65.4)**	10 (34.5)*	13 (26.5)*	40 (38.5)
Ocular history				
uveitis	1 (3.8)	2 (6.9)	3 (6.1)	6 (5.8)
ocular trauma	3 (11.5)	6 (20.7)	12 (24.5)	21 (20.2)
macular degeneration	1 (3.8)	1 (3.4)	6 (12.2)	8 (7.7)
glaucoma	2 (7.7)	4 (13.8)	6 (12.2)	12 (11.5)
diabetes	3 (12)	2 (6.9)	11 (22.4)	16 (15.5)
contralateral detachment	1 (3.8)	1 (3.4)	0 (0)	2 (1.9)

^**^p < 0.05

### Visual acuity

Overall, mean pre-operative visual acuity (logMAR) was 1.3 ± 0.65. While mean pre-op visual acuity did not differ among the patients of the different surgical timing groups (early, moderately delayed, late) (*p* = 0.62), all patients with light perception visual acuity at their pre-operative visit had late repair (detachment symptoms lasting ≥ 7 days) (*n* = 8.0, 17%) (*p* = 0.01). Mean post-operative visual acuity for each surgical timing group was calculated using measured visual acuity at 3-, 6-, and 12-months post-operative and final visit and shown in (Table 2).

**Table 2:** Visual acuity outcomes among patients with early, moderately delayed, and late fovea-involving RRD repair

	Mean visual acuity ± SD (logMAR)		
	Early	Moderately Delayed	Late
pre-op	1.4 ± 0.70	1.3 ± 0.59	1.3 ± 0.68
3 months	0.58 ± 0.62	0.59 ± 0.57	1.0 ± 0.67*
6 months	0.52 ± 0.74	0.48 ± 0.50	1.0 ± 0.79*
12 months	0.39 ± 0.53	0.71 ± 0.64	0.65 ± 0.62*
final visit	0.28 ± 0.43	0.35 ± 0.44	0.90 ± 0.79*

*Bonferroni *P*-Value < 0.05

Between-group analysis comparing surgical timing showed no difference in post-operative visual acuity between patients with detachments undergoing early versus moderately delayed repair of RRD (*p* = 0.163). However, mean visual acuity differed between patients undergoing early versus late repair (*p* = 0.010). Superior mean visual acuity was observed at 3-, 6-, and 12-months post-op visits in undergoing early compared to late repair (Bonferroni corrected *p*-values = 0.004, 0.013, and 0.012, respectively). Similarly, mean visual acuity differed between patients with moderately delayed versus late repair. Superior mean visual acuity was observed at 3 and 6 months in patients with moderately delayed repair compared to those with late repair (Bonferroni-corrected *p*-values: 0.006, and 0.006, respectively) (Table 2). The observed superior visual outcomes in early and moderately delayed repair of compared to late repair are maintained at final time-adjusted visual acuity measurements (Table 2).

Indicators of severe detachment such as the presence inferior or giant tears, intraoperative use of silicone oil, and extent of detachment in clock hours were evaluated as potential contributors and confounders to poor post-operative outcome. Of these variables, only the use of Silicone oil was found to be associated with worse mean visual acuity at final post-op visit with mean logMAR difference 0.67 for cases with vs without silicone oil use [95% CI 0.19—1.16] ( *p* = 0.01) (Table 3).

**Table 3:** Final visual acuity outcomes among patients with severe detachments (inferior tears, giant tears, SO use, and high detachment clock hour)

	sample (%)	mean final visual acuity ± SD (logMAR)	*p*-value
Inferior Tear			
N	68	0.55 ± 0.70	0.51
Y	31	0.65 ± 0.12	
Giant Tear			
N	51	0.58 ± 0.71	0.96
Y	48	0.58 ± 0.66	
Silicon Oil use			
N	91	0.53 ± 0.65	0.01
Y	8	1.2 ± 0.76	

### Intraocular pressure

Overall mean pre-operative IOP was 12.5 ± 5.81 with no significant difference in patients with different timings of repair (*p* = 0.664). Post-operative IOP did not significantly differ across 3, 6, and 12 months follow- up visits or final time-adjusted visit for patients within the same detachment timing group. Likewise, between group comparison of mean IOP throughout the follow-up period did not reveal any significant differences among patients with differing detachment repair timing (Table 4).

**Table 4:** Intraocular pressure (IOP) outcomes among patients with early, moderately delayed, and late fovea-involving RRD repair

	Mean IOP ± SD (mmHg)		
	Early	Moderately Delayed	Late
pre-op	12.7 ± 4.02	13.1 ± 6.25	11.9 ± 6.41
3 months	13.5 ± 5.73	15.9 ± 8.03	13.8 ± 5.24
6 months	14.0 ± 5.12	15.5 ± 4.56	14.1 ± 6.42
12 months	12.5 ± 4.05	15.1 ± 5.57	13.4 ± 6.70

### Complications

Re-detachment occurred in 23 (22%) of patients. 6 (26%) re-detachments occurred in the early repair group, 4 (17%) re-detachments occurred in the moderately delayed group, and 13 (56%) re-detachments occurred in the late repair group. No significant difference was observed in re-detachment rate among patients of different surgical timing groups (*p* = 0.44). Silicone oil was used in 8.6% of eyes that became redetachment (*n* = 2). There was no statistical association between re-detachment and case length (*p* = 0.847). Re-detachment was observed less frequently in patients receiving 360 laser (9.4%) compared to patients receiving focal laser (42.5%), OR 6.70 (95% CI 1.93- 23.2 (Table 5). Other surgical complications identified in this study include post-operative glaucoma (18%), CME (13%), ERM (27%), and cataract (36%) (Table 6). Analysis of these cases regarding timing of repair and factors indicative of severe detachment did not identify any predictive variables for post-op complications.

**Table 5:** Summary re-detachment rate statistics

Linear Regression Model for Re-detachment					
Predictor	Ratio		Odds Ratio [95% CI]	*p*-value	
Average case length (min)	X + 30 min:X		1.05 [0.640—1.71]	0.847	
*360 Laser usage*	No: Yes		6.70 [1.93—23.2]	0.003	
Re-detachment Rate among Surgical Timing groups					
Overall		23(22%)			Pearson Exact Chi-Square Test: *p* = .44
Early		6.0 (23%)			
Moderately Delayed		4.0 (14%)			
Late		13 (27%)

**Table 6:** Post-op complication rate among patients with varying surgical timing, inferior tear, and giant tear status

	Glaucoma *n* = 19 (18)	CME *n* = 13 (12.5)	ERM *n* = 29 (28)	Cataract *n* = 37 (36)
Surgical Timing Group				
Early	5.0 (19)	3.0 (12)	8.0 (31)	6.0 (23)
Moderately Delayed	7.0 (24)	3.0 (10)	9.0 (31)	15 (52)
Late	7.0 (14)	7.0 (14)	12 (25)	16 (33)
Pearson Exact Chi-Square Test (*P*-value)	0.52	0.93	0.75	0.08
Inferior Tear Status				
Y	8.0 (25)	7.0 (22)	9.0 (28)	10 (31)
N	11 (15)	6.0 (8.3)	20 (28)	27 (38)
Fisher Exact Test (*P*-value)	0.28	0.10	1.0	0.66
Giant Tear Status				
Y	11 (22)	7.0 (22)	12 (24)	12 (24)
N	8.0 (15)	6.0 (8.3)	17 (31)	25 (46)
Fisher Exact Test (*P*-value)	0.45	1.0	0.51	0.02

## Discussion

Our study has demonstrated that repair of macula involving RRD 3–7 days after symptom onset does not appear to have a significant disadvantage compared to repair within 48 h. Delaying repair of macula involving RRD until > 7 days does significantly impact visual outcomes. Additionally, the use of 360 laser was shown to be protective against re-detachment in our study cohort.

The current standard practice for retinal detachment surgical repair utilizes macula inclusion in detachment as a measurement for helping determine the urgency of repair required. This criterion was established through previous studies that concluded that functional damage with macula detachment appears permanent [1]. With this reasoning, macula-on retinal detachment has been treated as an emergency contrasting with the practice of waiting to repair foveal-involving RRD. Usage of this concept to determine repair urgency has been challenged as duration of detachment remains a key modifiable risk factor for outcomes of fovea-involving RRD repair [18, 19]. This study aimed to inform decisions of duration of time before 23-g PPV for repair of fovea-involving RRD via a retrospective analysis of visual acuity outcomes and complication rates. While IOP measurements and complication rates were similar across patients receiving repair at different times, better visual acuity measurements at 3-, 6-, and 12-month post-op points were correlate with a detachment to repair time shorter than 7 days.

Previous studies have found similar correlations with shorter duration of detachment and better visual acuity with follow up, suggesting that fovea-involving retinal detachment should not be postponed due to potential of worse long-term visual outcomes in patients, but the specific cut-off for surgical intervention remains unclear. Interestingly, our finding of similar visual outcomes among patients with reported detachment symptoms < 48 h and those with reported symptoms ≥ 48 h and < 7 days would suggest equivalent visual outcomes in early and moderately delayed repair and may justify a tolerable delay period of up to 1 week from onset of reported symptoms.

This finding is in line with a previous retrospective analysis with a mean follow-up period of 19.6 months which determined visual outcomes for repair at any point within the initial 7 days of detachment were similar [20]. In addition, another retrospective study demonstrated significantly better visual outcomes in patients with repairs done within the first three days, with no difference after 10 days [21]. A previous review of literature of 9 retrospective analyses of fovea-involving RRD repair via scleral buckle found optimal results within the first 72 h. Unfortunately, there was insufficient vitrectomy outcomes data to draw conclusions in this review [10]. As the specifically assessed timepoints of detachment remain varied among the literature, further study is needed to clarify this specific cutoff for duration of symptoms which represents a tolerable delay of surgical repair where visual outcomes remain optimal.

Of note, we also report a redetachment rate of 22% that is quite high in comparison the literature [22]. While not directly relevant to our study, it is unclear why re-detachment rates were so high in this study. We hypothesize our dataset may represent inherently more complicated retinal detachment repairs given the involvement of the fovea in all cases, high amount of observed inferior and giant tears, and nearly half of patients presenting with > 7 days symptoms.

As the aim of this study was to determine how emergently fovea-involving retinal detachments should be addressed, we chose to split timing of repair into early, moderately delayed, and late categories, despite up to 47% of total cases falling within the > 7 days symptom category. This group was proportionately the most largely represented group in our analysis with mean duration of 32.4 days (range 10–180 days). Potentially, visual repair at 8 days of symptom onset may differ from repair at 3 months, limiting interpretation of our results from this group. However, such conclusions fall outside the scope of this study which aims to define the early window for optimal visual recovery, and further work may better characterize the effect of late surgical repairs specifically.

The effect of surgical modality for early, moderately delayed, or late repair of rhegmatogenous retinal detachment is also not addressed in this study which sought to evaluate the effect of only surgical timing on visual outcome. Cases repaired with scleral buckle, pneumatic retinopexy, or combination scleral buckle with vitrectomy were excluded. Additionally, as our attending surgeon practice patterns are to preferentially repair rhegmatogenous retinal detachments with 23-gauge vitrectomy, 25- and 27-gauge vitrectomy cases were excluded given the relative paucity of cases. Future work may better elucidate whether repair modality should influence decisions to delay fovea-involving rhegmatogenous detachment repair.

Limitations of this study include its retrospective design, exclusive use of 23-g vitrectomy, reliance on patient reporting for macular detachment, and potential confounding variables effecting post-operative visual acuity. As previously discussed, while most pre-existing literature regarding the timing of surgical repair of RRD have utilized similar methodologies for evaluating duration of retinal detachment, the assessment of fovea-involving RRD duration does not have standardized objective criteria. Instead fovea-involving RRD duration relies on patient reporting their symptoms as a proxy measurement for the duration of detachment. Therefore, in some patients a mismatch between symptoms duration and timing of true detachment is plausible and confounds interpretation of surgical timing outcomes. This may be further complicated in the 8 patients with pre-operative ARMD in this study as the presence of baseline macular disease may hinder accurate symptomatic identification of macular detachment. Interestingly, one analysis has suggested up to a third of patients with assumed fovea attachment based on clinical symptoms and exam were shown to have true detachment on OCT [23]. Due to surgeon preference at our institution, there were only a small number of 25-g repairs available for review which were subsequently excluded for the analysis. 25-g vitrectomy has recently gained popularity due to the larger wound required for 23-g vitrectomy and more vitreous reflux and incarceration in the cannula compared to 25-G systems [24–26]. Incidence of certain complications with 23-g systems may also be higher including significant subconjunctival hemorrhage, sclerotomy leakage, postoperative hypotony, choroidal detachment, and exogenous endophthalmitis although we did not observe any of these complications in our retrospective review [27, 28].

This study also does not evaluate various operative, and postoperative factors which may influence the visual acuity results observed in this study including use of silicone oil as a tamponade agent in initial repair, removal of silicone oil, post-op cataract surgeries, YAG capsulotomies, ERM removals, and treatment of post-op macular edema. It is plausible that patient visual acuity at 3 or 6 months may not represent a fully completed post-operative treatment due to the reasons above and therefore not a stable visual acuity measurement. Finally, follow up time was quite variable in this study (mean 17.9 ± 14.1 months), longer and more comprehensive follow-up data may demonstrate further differences in visual outcomes based on time from detachment to repair.

## Conclusions

In our study evaluating the effect of surgical timing for repair of primary fovea-involing rhegmatogenous retinal detachment, visual outcomes appear similar for patients undergoing early repair within 48 h and those undergoing moderately delayed repair 3–7 days from symptom onset. Delaying surgery beyond 7 days from symptom onset was associated with worse visual recovery. 360 laser appeared protective against retinal detachment overall.

## Supplementary Information

**Additional file 1:** **Supplemental Table 1. **Tamponade Agent used in early, moderate, and delayed fovea-involving RRD repair

## Data Availability

All data generated or analyzed during this study are included in this published article.

## Abbreviations

RRD: Rhegmatogenous retinal detachment
23-g: 23-Gauge
PPV: Pars plana vitrectomy
OCT: Optical coherence tomography
IOP: Intraocular pressure
CF: Count fingers
HM: Hand motion
LP: Light perception
NLP: No light perception
