# Supplementary material for: Effect of surgical timing in 23-g pars plana vitrectomy for primary repair of macula-off rhegmatogenous retinal detachment, a retrospective study
Source: BMC Ophthalmol. 2022 Mar 25;22:136. doi: 10.1186/s12886-022-02364-4 (PMC8957134; doi:10.1186/s12886-022-02364-4)
Supplement: Supplementary file 1 — Additional file 1: Supplemental Table 1. Tamponade Agent used in early, moderate, and delayed fovea-involving RRD repair [file 12886_2022_2364_MOESM1_ESM.docx]

**Supplemental Table 1: Tamponade Agent used in early, moderate, and delayed fovea-involving RRD repair**

|  | Early | Moderately Delayed | Late | Total |
| --- | --- | --- | --- | --- |
| Filtered Air | 5 | 6 | 19 | 30 |
| SF6 | 4 | 5 | 2 | 11 |
| C3F8 | 13 | 17 | 19 | 49 |
| Silicone | 3 | 1 | 9 | 13 |
| Total | 26 | 29 | 49 |  |
